# Investigating the lasting effects of SARS-CoV-2 infection and the lung microbiome: no persistent microbial alterations in recovered COVID-19 patients with persistent radiological or respiratory abnormalities

**DOI:** 10.1099/acmi.0.001103.v3

**Published:** 2026-06-22

**Authors:** Nancy M.Y. Teng, Bavithra Vijayakumar, David J.F. Smith, James Tonkin, Christopher M. Orton, Justin L. Garner, James A. Harker, Clare M. Lloyd, Philip L. Molyneaux, Pallav L. Shah

**Affiliations:** 1 National Heart and Lung Institute, Imperial College London, London, UK; 2 Chelsea and Westminster Hospital, London, UK; 3 Royal Brompton and Harefield Hospitals, Guy’s and St Thomas’ NHS Foundation Trust, London, UK; 4 Imperial College Healthcare NHS Trust, London, UK; 5 Asthma UK Centre for Allergic Mechanisms of Asthma, London, UK

**Keywords:** 16S rRNA gene amplicon sequencing, airway microbiota, coronavirus disease 2019 (COVID-19), microbiota, pulmonary fibrosis

## Abstract

Severe acute respiratory syndrome coronavirus 2 (SARS-CoV-2) was a global pandemic where infected individuals experienced mild or severe disease. Unfortunately, some patients who experienced severe disease also had lasting abnormalities. The lung microbiome of 38 adult coronavirus disease 2019 (COVID-19) patients with persistent respiratory symptoms and/or radiological abnormalities was analysed. The aim was to investigate whether the lasting radiological abnormalities reported in this cohort were associated with an altered airway. Thirty-six bronchoalveolar lavage fluid samples from patients underwent 16S rRNA gene amplicon sequencing and were compared to 28 non-fibrotic control samples from a previously published study. COVID-19 patients had statistically significantly greater number of genera but at uneven abundances, though not statistically significant compared to non-fibrotic controls. Permutational ANOVA (PERMANOVA) suggested that COVID-19 can influence the lung microbiome composition after accounting for multivariate dispersion. Further analysis showed differences in the relative abundances of *Actinomyces*, *Neisseria*, *Haemophilus*, *Rothia* and *Gemella*. Indicator species analysis showed that a COVID-19 lung microbiome profile could be driven in part by differences in *Fusobacterium*, *Actinomyces*, *Catonella*, *Oribacterium* and *Mycobacterium*. Associations with clinical parameters were lacking apart from CT lung opacification, which revealed a significant negative association with the number of genera. Differential abundance analysis with MaAsLin2 pointed towards *Porphyromonas* as a potential explaining genus, though this was not significant after post hoc corrections. DESeq2 revealed enriched oral taxa in the BAL samples, suggesting potential oral-translocation reflective of a disease state. Our findings suggest that individuals with persistent radiological abnormalities following SARS-CoV-2 infection have experienced subtle shifts in their microbiome profile, but these are not strongly associated with clinical phenotypes and, therefore, unlikely of significance.

## Data Summary

All fastq files used in this study are available on National Center for Biotechnology Information (NCBI) using the BioProject accession numbers presented in Table 1.

**Table 1.** Fastq files accession numbers available on NCBI

PRJNA1299772 (COVID-19)	PRJNA1109293 (reagent controls)	PRJNA609242 (non-fibrotic controls)
SAMN50314802	SAMN41260967	SAMN15103672
SAMN50314803	SAMN41260966	SAMN15103749
SAMN50314804	SAMN41260965	SAMN15103754
SAMN50314805	SAMN41260964	SAMN15103761
SAMN50314806	SAMN41260963	SAMN15103770
SAMN50314807	SAMN41260962	SAMN15103771
SAMN50314808	SAMN41260961	SAMN15103773
SAMN50314809	SAMN41260960	SAMN15103786
SAMN50314810	SAMN41260959	SAMN15103787
SAMN50314811	SAMN41260958	SAMN15103788
SAMN50314812		SAMN15103789
SAMN50314813		SAMN15103675
SAMN50314814		SAMN15103790
SAMN50314815		SAMN15103791
SAMN50314816		SAMN15103792
SAMN50314817		SAMN15103793
SAMN50314818		SAMN15103794
SAMN50314819		SAMN15103795
SAMN50314820		SAMN15103796
SAMN50314821		SAMN15103797
SAMN50314822		SAMN15103798
SAMN50314823		SAMN15103677
SAMN50314824		SAMN15103683
SAMN50314825		SAMN15103686
SAMN50314826		SAMN15103688
SAMN50314827		SAMN15103691
SAMN50314828		SAMN15103733
SAMN50314829		SAMN15103746
SAMN50314830		
SAMN50314831		
SAMN50314832		
SAMN50314833		
SAMN50314834		
SAMN50314835		
SAMN50314836		
SAMN50314837		
SAMN50314802		
SAMN50314803		
SAMN50314804		

## Introduction

Severe acute respiratory syndrome coronavirus 2 (SARS-CoV-2) led to the global pandemic in 2020. Infected patients experienced anything from mild coryzal symptoms to severe respiratory distress and a minority were left with residual pulmonary fibrosis [1,2]. It remains unknown why some patients experience residual pulmonary fibrosis whilst others do not. Adult patients recovered from SARS-CoV-2 infection, hereafter referred to as coronavirus disease 2019 (COVID-19), discharged between March and June 2020, were prospectively assessed at 3 months and 1 year in the PHENOTYPE study [3], with evaluations including symptoms, functional impairment and thoracic CT scans. Whilst significant proteomic and immune shifts characterize these persistent abnormalities, such changes may be downstream effects of a more fundamental lung dysbiosis. The lung microbiome, long overlooked due to the historical assumption of lung sterility, has emerged as a key regulator of local mucosal immunity [4]. Given its ability to chronically stimulate inflammatory pathways, the microbiome represents a candidate for explaining the immune activation observed in these patients. Recent studies have confirmed strong associations between airway microbial communities and various chronic disease states [5,8]. Therefore, we investigated whether the lung microbiome could provide an explanation for the persistent respiratory abnormalities following COVID-19 infection. From the ‘PHenotyping patiENts Admitted to Hospital With cOvid-19 Infection and idenTifYing Prognostic markErs’ (PHENOTYPE, NCT04459351) study, a subset of 38 patients with persistent radiological abnormalities underwent diagnostic bronchoscopy. Peripheral blood and bronchoalveolar lavage (BAL) samples were collected for research. BAL samples underwent 16S rRNA gene amplicon sequencing to investigate if the airway microbiome could explain the presence of persistent respiratory abnormalities.

## Method

### Study design and ethics

Information with regard to demographics of the patient cohort and recruitment process can be found in Vijayakumar *et al*. [3,9]. Briefly, this study included BAL samples from the PHENOTYPE study (NCT: 04459351, IRAS: 284497 from the Yorkshire and The Humber – Sheffield Research Ethics Committee). Patients from the Chelsea and Westminster Hospital in London, who were over 18 years of age, had a previous confirmed diagnosis of COVID-19 by antibody or positive PCR and experienced persistent respiratory symptoms that necessitated follow-up outpatient respiratory appointments, were asked to enrol in the study. Patients underwent bronchoscopy if they presented with abnormal CT findings at 3 months post-discharge from the hospital. Patients with abnormal CT findings and under sedation had 150 ml of sterile saline flushed through the most affected area, as determined by the CT.

### DNA sequencing and post-sequencing processing

Genomic DNA was extracted from BAL samples and sequenced using 16S rRNA gene amplicon sequencing, targeting the V4 hypervariable region, as previously described in [10]. Ten reagent controls were also processed alongside the samples. Briefly, genomic DNA was extracted from BAL pellets using phenol–chloroform methods [7,10]. The V4 hypervariable region of the 16S rRNA gene was sequenced using primer sequences 515F (5′-GTGCCAGCMGCCGCGGTAA-3′) and 806R (5′-GGACTACHVGGGTWTCTAAT-3′) and sequenced using Illumina MiSeq PE150 chemistry. We were unable to collect contemporaneous non-fibrotic controls due to the ongoing pandemic, so we used samples from non-fibrotic controls from a previous study by Invernizzi *et al*. [7]. These samples were extracted and processed identically and raw sequence reads were used in a combined analysis as outlined below. Sampling controls were included and processed in tandem [10].

Sequences were denoised, filtered and assigned taxonomy using qiime2 [11]. Sequences were trimmed by 15 nt and truncated to 140 bp before denoising using the default parameters from DADA2 [12]. Denoised sequences were aggregated into amplicon sequence variants and classified using the Green Genes 13_8_otus database downloaded in November 2023. Taxa below a minimum abundance of 0.005% [13] were removed, thereby removing most of the environmental contaminants. The final dataset used for analysis had a total number of 3,568,484 reads, with a median of 32291.5 reads per sample. R version 4.1.2 was used for subsequent analysis with packages: phyloseq-v1.38.0 [14], qiime2R-v0.99.6 and Decontam-v1.14.0 [15] used for quality processing. For data analysis, taxa were aggregated at the genus level, ignoring clade-level distinction that is given by Greengenes2. Microbiome analysis was performed using vegan-v2.6.10 [16], MaAsLin2 [17] and DESeq2 [18], and data were visualized using ggplot2-v3.5.2.

### Bacterial burden

Bacterial burden was determined using droplet digital PCR. The primers used for this were 5′-GCAGGCCTAACACATGCAAGTC-3′ (63F) and 5′-CTGCTGCCTCCCGTAGGAGT-3′ (355R) [19,20]. The BAL samples were compared to negative controls, which were bronchoscope flushes. This was a saline solution flushed through the scope prior to collecting the BAL fluid for research. This was done to capture contaminants introduced by the scope or environment.

### Clinical metadata criteria

CT scans were reviewed by two thoracic radiologists with over 20 years of experience. They were blinded to clinical data prior to review. Scans were scored via consensus, and the overall extent of opacified lung was rounded to the nearest 5%. Blood results reported for fibrinogen, C-reactive protein, ferritin, lymphocytes and neutrophils are the values highest during admission.

### Code availability

The code and sample IDs used for the analysis in this study are available on https://github.com/molyneaux-lab. The 16S rRNA gene amplicon sequences were deposited with the BioProject accession number: PRJNA1299772.

## Results

Clustering of samples using UniFrac weighted distances on a principal component analysis (PCoA) showed six samples clustered separately (Fig. S1A, available in the online Supplementary Material), likely due to environmental contamination. Visualizing the relative abundances of the top ten most abundant genera identified, *Sphingomonas* being the main contributor of these six samples clustering separately (Fig. S1B). *Sphingomonas* is known as a contaminant of low-biomass samples; therefore, these samples were removed from downstream analysis.

### Alpha and beta diversity

**Fig. 1.**
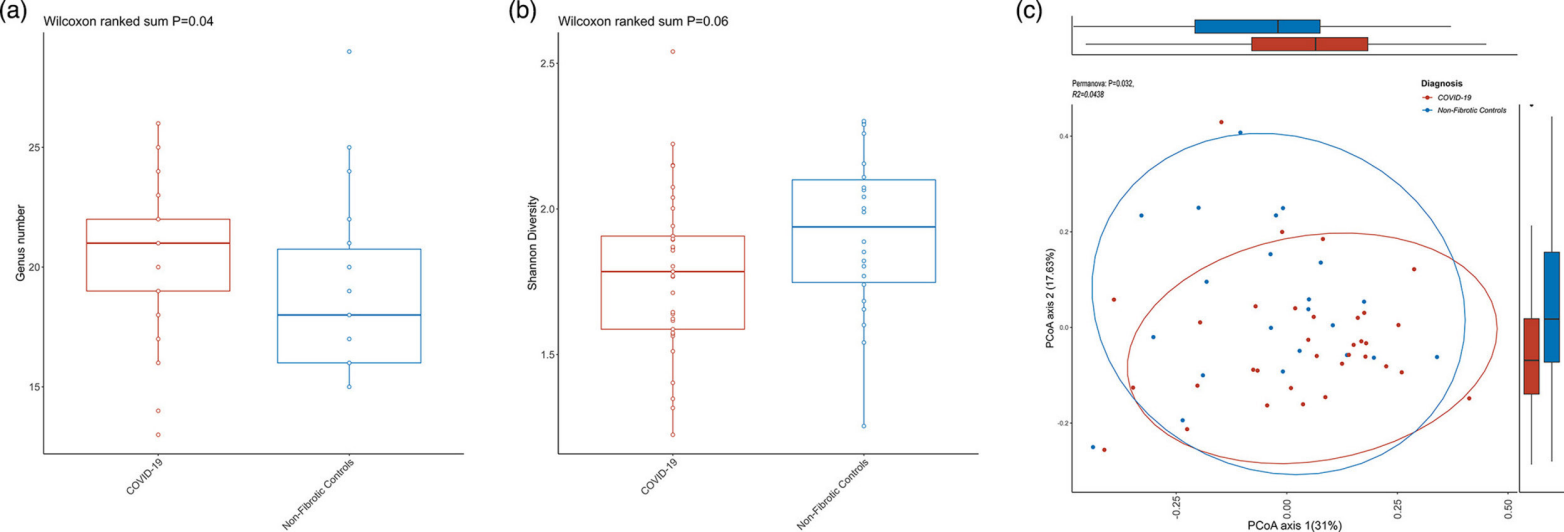
There are subtle differences in the microbiome of COVID-19 patients compared to non-fibrotic controls. (**a**) COVID-19 patients showed a higher number of unique genera (Wilcoxon ranked sum, *P*=0.04). (**b**) Airway microbiome communities were less even in COVID-19 patients, but this did not reach statistical significance (Wilcoxon ranked sum, *P*=0.06). Beta diversity visualized by PCoA using Bray–Curtis dissimilarity shows segregation between the lung microbiome of COVID-19 compared to non-fibrotic controls (PERMANOVA, *P*=0.035).

Genus number was higher in COVID-19 patients than the non-fibrotic control group (Wilcoxon ranked sum, *P*=0.04, Fig. 1a). Shannon diversity index, a reflection of richness whilst accounting for abundances, was lower in COVID-19 (Wilcoxon ranked sum, *P*=0.06, Fig. 1b) but not significant. Taken together, it suggests that the lung microbiome of COVID-19 patients may have more bacterial members at an uneven abundance compared to non-fibrotic controls, but this was not significant in our cohort. Beta diversity was assessed using PCoA and Bray–Curtis dissimilarity, and permutational ANOVA (PERMANOVA) was used to assess if their diagnosis could explain the microbial composition. PERMANOVA was significant (Fig. 1c, *P*=0.035), and segregation between the communities can be seen on both axes. To account for dispersion effects of the BAL samples, we also performed dispersion PERMANOVA, which was not significant (*P*=0.933), suggesting that the airway microbiome of COVID-19 patients experiences a compositional shift compared to non-fibrotic controls.

### Composition of the airway microbiome

**Fig. 2.**
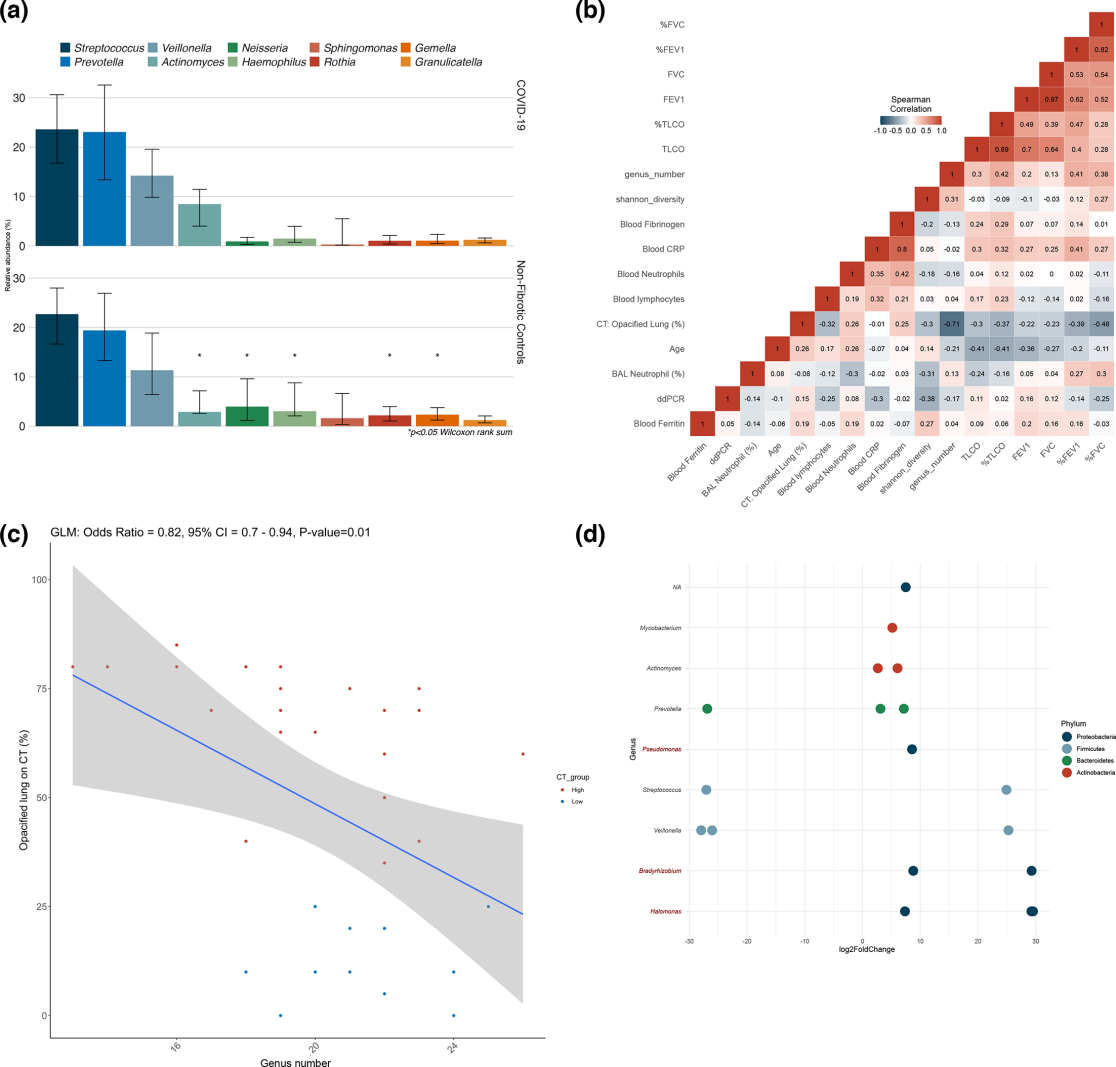
Differences in the microbiome do not explain clinical outputs. (**a**) Several genera were lower in COVID-19 patients compared to non-fibrotic controls (Wilcoxon-ranked sum, *P<0.05). Data are presented as median with error bars denoting inter-quartile range. (**b**) Spearman’s correlation revealed correlations amongst pulmonary lung function tests, i.e. TLCO, %TLCO, FEV1, FVC, %FEV1, %FVC, but not with other parameters. (**c**) General linear modelling with opacified lung on CT scans revealed a negative relationship between opacification and genus number (*P*=0.01). CT_group: high ≥25% opacification or low <25% opacification. (**d**) DESeq2 analysis revealed several taxa enriched in the COVID-19 microbiome, including some environmental bacteria annotated in red. DESeq2 analysis was relative to non-fibrotic controls, and the *P*-value threshold was set to p-adjusted=0.01. TLCO, transfer factor of the lung for carbon monoxide; %TLCO, normalized TLCO; FEV1, forced expiratory volume in one second; %FEV1, normalized FEV1; FVC, forced vital capacity; %FVC, normalized FVC; CRP, C-reactive protein.

We next assessed the top ten most abundant genera in the microbiome of COVID-19 and non-fibrotic control groups. *Neisseria*, *Haemophilus*, *Rothia* and *Gemella* were significantly higher (Wilcoxon–ranked sum, *P*<0.05) in non-fibrotic control patients, and *Actinomyces* was higher in COVID-19 patients (Fig. 2a). This confirms our earlier observations (Fig. 1b) where the airway microbiome of COVID-19 patients had greater unevenness compared to non-fibrotic controls. Indicator species analysis also suggested that *Fusobacterium*, *Actinomyces*, *Catonella* and *Mycobacterium* are associated with the lung microbiome of COVID-19 patients, whilst non-fibrotic control patients have *Rothia* and *Actinobacillus* associated with their lung microbiomes. These results suggest that there are some taxonomical differences between COVID-19 patients with lasting respiratory abnormalities and non-fibrotic controls. However, a statistically significant effect size may be nuanced.

### Microbiome associations with clinical parameters

Droplet digital PCR was performed to quantify bacterial burden. This metric has previously been reported with worse survival in pulmonary fibrosis [6,8]. In our cohort, bacterial burden did not differ between the COVID-19 and non-fibrotic control groups (Wilcoxon ranked sum, *P*=0.65). When looking into the COVID-19 severity classification, there was no association between bacterial burden and severity of disease (Kruskal–Wallis, *P*=0.8). Pearson correlations of clinical parameters showed strong associations with pulmonary functional outputs, but not with blood markers, features of the microbiome nor bacterial burden (Fig. 2b). However, it was noted that ‘CT: opacified lung (%)’ was negatively correlated with genus number. Generalized linear modelling with a quasibinomial family and logit link was used to assess the association between genus number and CT opacification (>25%), adjusting for age, sex and smoking status. This revealed that higher species number was associated with a decrease in odds of lung opacification on CT scans, after adjusting for sex, age and smoking status (Fig. 2c). Using the cut-off value of 25%, we grouped patients into high [CT: opacified lung (%) ≥25%] and low CT groups [CT: opacified lung (%) <25%]. Differential abundance analysis using MaAsLin2 suggested *Porphyromonas* being associated with low CT opacification, though this did not reach statistical significance (adjusted *P*-value=0.17). To account for the sparse nature of microbiome data, we also employed DESeq2, which offers greater sensitivity for the modest sample size of this cohort [18]. Taking these conservative approaches allowed us to detect subtle but potentially biologically relevant shifts that may be otherwise overlooked. DESeq2 analysis revealed several taxa that were shown to be enriched in the COVID-19 lung microbiome, including *Mycobacterium*, *Actinomyces*, *Prevotella*, *Pseudomonas*, *Streptococcus*, *Veillonella*, *Bradyrhizobium* and *Halomonas*.

## Discussion

In our analysis of BAL samples from recovered COVID-19 patients with persistent respiratory abnormalities, we observed subtle shifts in the airway microbiome. There was an increased number of genera in COVID-19 microbiome communities at uneven abundances, though not reaching statistical significance. Permanova indicated that COVID-19 microbiome communities do experience a shift from the non-fibrotic control communities. There were more nuanced statistically significant changes between these two groups, where *Neisseria*, *Haemophilus*, *Rothia* and *Gemella* were higher in the non-fibrotic control group, whilst *Actinomyces* was higher in COVID-19 patients. We observed the trend of higher species number being correlated with a lower lung opacity on CT scans. This is reflective of lower bacterial burden being correlated with slower disease progression being reported in IPF [6]. Though not reaching statistical significance, MaAsLin2 revealed *Porphyromonas* to be higher in the low lung opacity CT group. *Porphyromonas* is a known core respiratory microbiome member but remains understudied within its role in health and disease. It is speculated to be a pathobiont or passive member of the microbiome [21,22]. Although we observed an association between *Porphyromonas* and CT lung opacification, this did not reach statistical significance after post-hoc correction; this observation could be hypothesis-generating in future larger, longitudinal studies. DESeq2 analysis identified an enrichment of several taxa in COVID-19-recovered microbiomes, including *Streptococcus*, *Actinomyces*, *Prevotella* and *Veillonella*. These are traditionally considered oral cavity commensals [23]. Given the nature of BAL collection, we cannot definitively distinguish whether the enrichment of these oral-associated taxa represents a true shift in the lower respiratory tract, potentially driven by micro-aspiration or altered lung microbiome, or reflects procedural carry-over from the upper airways during bronchoscopy. However, it is noteworthy that Meng *et al.* did report enrichment of *Streptococcus* and *Veillonella*, appearing with co-infections in their COVID-19 cohort [24]. These findings may suggest that the post-COVID-19 lung environment may be more susceptible to micro-aspirations or microbial translocation from the oral cavity to the lower respiratory tract. Whilst we cannot rule out topological carry-over, the recurrence of these taxa across an independent cohort suggests they may represent a biological signature of the post-COVID airway.

Whilst our findings are intriguing and there are some significant findings observed, the modest number of samples may have restricted the detection power of finding associations of the lung microbiome within this cohort. To compare our COVID-19 cohort with controls, we opted to use non-fibrotic control samples from another study [7]. This does introduce the possibility of a batch effect. These samples were processed with the same extraction protocol, sequencing technology and sequencing protocol as the PHENOTYPE BAL samples. Raw FASTQ files of the control group were downloaded and processed with the PHENOTYPE samples to ensure that post-sequencing processing was consistent. We have opted to choose these samples as a comparator to provide context for these findings. Although we took measures to account for batch effect due to the nature of this study design, it cannot be fully eliminated. In our data analysis, we have chosen a threshold filtering level of 0.005% for our study following the conservative suggestion published by Bokulich *et al*. [13]. To maintain the compositional integrity of the dataset and avoid the inflation of relative abundances (type I error), identified contaminants were not physically removed from the main analysis. Instead, we used the decontam package [15] to rigorously identify contaminants using reagent controls. Of the 17 identified contaminants, 16 were ranked 196th or lower in total abundance, indicating minimal impact on community structure. While one contaminant was ranked 17th (*Bradyrhizobium*), its presence was consistent across groups and had a total abundance of 0.009% (Fig. S2). By retaining these taxa to preserve compositional proportions, our differential abundance models identified several significant features that likely represent environmental noise. Specifically, *Halomonas* [10] and *Bradyrhizobium* [25] were identified by decontamination (Table S1), whilst *Pseudomonas* [25,26] is recognized in literature to be a contaminant of low-biomass samples. The presence of these signatures in COVID-19 BAL samples may reflect the challenges of sample collection in a high-pressure clinical setting or a subtle shift in the low-biomass lung environment. We present these findings transparently, acknowledging the trade-off between strict contaminant removal and the preservation of compositional data structure.

Taken together, the absence of significantly different community structures and lack of associations with clinical parameters led us to conclude that the lung microbiome of COVID-19 patients with persistent parenchymal abnormalities does not differ significantly from that of non-fibrotic control patients. There may be nuanced differences that could suggest an altered state being associated with residual pulmonary fibrosis experienced by these patients, but larger studies need to be conducted to achieve this [9]. Our findings align with the recent work of Smith *et al.*, who observed that microbiome profiles in post-COVID-19 patients largely resemble those of non-fibrotic controls. When viewed alongside Meng *et al.*, who reported distinct shifts during active infection compared to recovery, a trend can be seen. Whilst COVID-19 can trigger an acute microbial dysbiosis, the respiratory tract appears to revert towards a homeostatic, healthy-like profile during recovery irrespective of radiological abnormalities. This suggests that the persistent radiological abnormalities observed in post-COVID fibrosis may be driven by host-pathway injury rather than ongoing microbial dysbiosis.

## Supplementary material

10.1099/acmi.0.001103.v3 Supplementary Material 1.

## Abbreviations

BAL: bronchoalveolar lavage
COVID-19: coronavirus disease 2019
NCBI: National Center for Biotechnology Information
PCoA: principal component analysis
PERMANOVA: permutational ANOVA
