# Supplementary material for: Investigating the lasting effects of SARS-CoV-2 infection and the lung microbiome: no persistent microbial alterations in recovered COVID-19 patients with persistent radiological or respiratory abnormalities
Source: Access Microbiol. 2026 Jun 22;8(6):001103.v3. doi: 10.1099/acmi.0.001103.v3 (PMC13286284; doi:10.1099/acmi.0.001103.v3)
Supplement: Supplementary Material 1. [file acmi-8-01103-s001.pdf]

Investigating the lasting effects of SARS-CoV-2 infection and the lung microbiome: No persistent microbial alterations in recovered COVID-19 patients with persistent radiological or respiratory abnormalities.

Nancy MY Teng[1,7], Bavithra Vijayakumar[1,2,3,7], David JF Smith[1,3], James Tonkin[2,3,4], Christopher M Orton[1,2,3], Justin L Garner[2,3,4], James A Harker[1,5], Clare M Lloyd[1,5], Philip L Molyneaux[1,3], Pallav L Shah [1,2,3,8]

[1]National Heart and Lung Institute, Imperial College London, London, UK
[2]Chelsea and Westminster Hospital, London, UK
[3]Royal Brompton and Harefield Hospitals, Guy's and St Thomas' NHS Foundation Trust, London, UK
[4]Imperial College Healthcare NHS Trust
[5]Asthma UK Centre for Allergic Mechanisms of Asthma, London, London, UK
[7]These authors contributed equally
[8] Corresponding author

Corresponding author

Pallav L Shah, National Heart and Lung Institute, Imperial College London, United Kingdom. pallav.shah@imperial.ac.uk

Supplementary materials

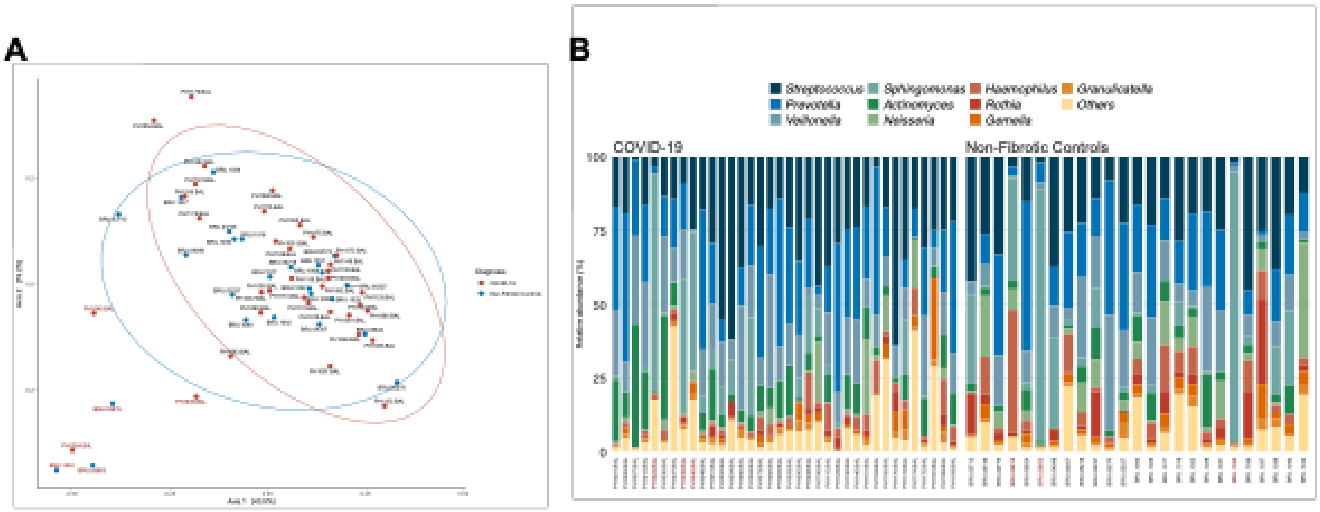

Supplementary figure 1: Removal of contaminated samples. (A) PCoA of weighted UniFrac distances identified six BAL samples clustering separately. (B) These samples had higher relative abundances of the known contaminant *Sphingomonas*. The six samples identified as being heavily contaminated are highlighted in red.

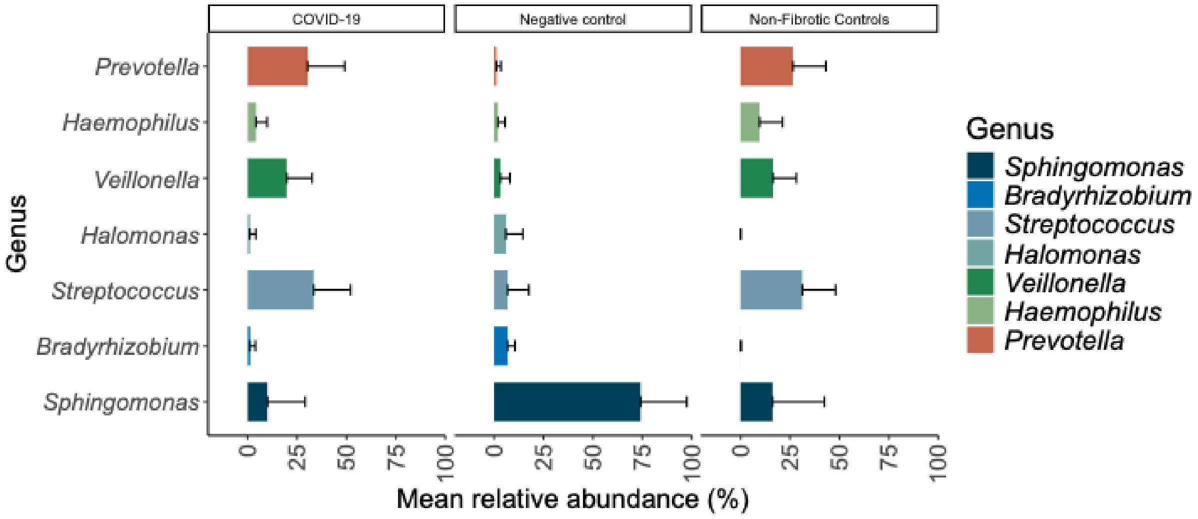

Supplementary figure 2: Relative abundances of identified contaminants using decontam. To provide transparency regarding potential noise; taxa identified as contaminants with a total abundance >0.01% are visualized. We observed that several identified taxa appear at low levels in negative controls but may represent indigenous respiratory microbiota. To preserve the compositional integrity of the dataset and avoid the introduction of Type I errors (false positives) through the artificial inflation of remaining taxa, these sequences were retained in the primary analysis.

Supplementary table 1: Identified ASV contaminants by decontam. Contaminant taxa were identified using ddPCR values and the "prevalence" method.

ASV	Family	Genus
102adec1a05d8f2be15e62ed6902edb7	*[Weeksellaceae]*	*Cloacibacterium*
f209b0b56491adc22f2df6336d6ca097	*Lachnospiraceae*	*Catonella*
c2d41dc0a7b8eaedcf4697512aee4427	*Staphylococcaceae*	*Staphylococcus*
36faca38e62a8e280ac588a2bd4c1eaf	*Sphingomonadaceae*	*Novosphingobium*
5c78314ff92e6fec9aa07acc1fa0dc24	*Bradyrhizobiaceae*	*Bradyrhizobium*
2aa46588194bc11bf193ea67ce5b00e2	*Bradyrhizobiaceae*	*Bradyrhizobium*
308ef02ccc5dc71986759531b6f4b6ed	*Corynebacteriaceae*	*Corynebacterium*
13862f192552f2fad61e3c092e9876c1	*Corynebacteriaceae*	*Corynebacterium*
7b67c5a74bac335a33ae9af0956f3f7c	*Corynebacteriaceae*	*Corynebacterium*
789510d034e52a5ee4db834a94ed5d76	*Mycobacteriaceae*	*Mycobacterium*
dd93bc698bdb8d4a9caa83e404af4bb5	*Micrococcaceae*	*Micrococcus*
3f6f8dbbfec63524058f6e15592bff13	*Halomonadaceae*	*Halomonas*
1bcbeecf21140f6de6151e34713a1801	*Halomonadaceae*	*Halomonas*
161213db112ce2246e2f09be596534f9	*Halomonadaceae*	*Halomonas*
74fc5efeb4b65ab769e7a99d184dff55	*Moraxellaceae*	*Acinetobacter*
4bccbdb96fb331b5bd8aec33cbb8a34e	*Moraxellaceae*	*Acinetobacter*
04a75f1199d1bc6b58d19dd8aeb24520	*Moraxellaceae*	*Enhydrobacter*
83b0bde4963afa2035706cc3fac899e7	*Oxalobacteraceae*	*NA*
17f016e3298748a0eb03b67eb9267a19	*Enterobacteriaceae*	*NA*
